# Small Molecule-facilitated Degradation of ANO1 Protein: A NEW TARGETING APPROACH FOR ANTICANCER THERAPEUTICS

**DOI:** 10.1074/jbc.M114.549188

**Published:** 2014-03-05

**Authors:** Anke Bill, Michelle Lynn Hall, Jason Borawski, Catherine Hodgson, Jeremy Jenkins, Philippe Piechon, Oana Popa, Christopher Rothwell, Pamela Tranter, Scott Tria, Trixie Wagner, Lewis Whitehead, L. Alex Gaither

**Affiliations:** From the ‡ Novartis Institutes for Biomedical Research, Cambridge, Massachusetts 02139,; the § Novartis Institutes for Biomedical Research, Horsham, West Sussex, RH12 5AB, United Kingdom, and; the ¶ Novartis Institutes for Biomedical Research, Basel CH-4002, Switzerland

**Keywords:** Anticancer Drug, Cancer Biology, Cancer Therapy, Chloride Channels, Computational Biology, ER-associated Degradation, Ion Channels, Molecular Modeling, Protein Degradation, Small Molecules

## Abstract

ANO1, a calcium-activated chloride channel, is highly expressed and amplified in human cancers and is a critical survival factor in these cancers. The ANO1 inhibitor CaCC_inh_-A01 decreases proliferation of ANO1-amplified cell lines; however, the mechanism of action remains elusive. We explored the mechanism behind the inhibitory effect of CaCC_inh_-A01 on cell proliferation using a combined experimental and *in silico* approach. We show that inhibition of ANO1 function is not sufficient to diminish proliferation of ANO1-dependent cancer cells. We report that CaCC_inh_-A01 reduces ANO1 protein levels by facilitating endoplasmic reticulum-associated, proteasomal turnover of ANO1. Washout of CaCC_inh_-A01 rescued ANO1 protein levels and resumed cell proliferation. Proliferation of newly derived CaCC_inh_-A01-resistant cell pools was not affected by CaCC_inh_-A01 as compared with the parental cells. Consistently, CaCC_inh_-A01 failed to reduce ANO1 protein levels in these cells, whereas ANO1 currents were still inhibited by CaCC_inh_-A01, indicating that CaCC_inh_-A01 inhibits cell proliferation by reducing ANO1 protein levels. Furthermore, we employed *in silico* methods to elucidate novel biological functions of ANO1 inhibitors. Specifically, we derived a pharmacophore model to describe inhibitors capable of promoting ANO1 degradation and report new inhibitors of ANO1-dependent cell proliferation. In summary, our data demonstrate that inhibition of the channel activity of ANO1 is not sufficient to inhibit ANO1-dependent cell proliferation, indicating that the role of ANO1 in cancer only partially depends on its function as a channel. Our results provide an impetus for gaining a deeper understanding of ANO1 modulation in cells and introduce a new targeting approach for antitumor therapy in ANO1-amplified cancers.

## Teaser

**Background:** The calcium-activated chloride channel ANO1 is highly expressed in cancer.

**Results:** Inhibition of ANO1 activity alone is not sufficient to inhibit cancer cell proliferation, suggesting a novel function of ANO1 protein in cancer.

**Conclusion:** The ANO1 inhibitor CaCC_inh_-A01 inhibits cancer cell proliferation by facilitating degradation of ANO1.

**Significance:** Our results may provide a new targeting approach for antitumor therapy in ANO1-amplified cancers.

## Introduction

Transformation of a normal cell to a cancer cell requires the acquisition of genetic abnormalities. Cancer cells become addicted to these driving factors, providing promising targets for therapeutic intervention (1). ANO1 has been shown to be amplified and highly expressed in several human carcinomas, including gastrointestinal stromal tumors, head and neck squamous carcinoma (HNSCC),^3^ esophageal squamous carcinoma (ESCC), and breast cancer (2–7). The coding sequence of ANO1 is located within the 11q13 amplicon, a genomic region that is frequently amplified in various human cancers, such as HNSCC, ESCC, gastrointestinal stromal tumors, and breast and bladder cancer. Overexpression and amplification of ANO1 in cancer had been described long before its molecular identity and function was discovered, resulting in multiple nomenclatures for ANO1 (also known as TMEM16A, DOG1, ORAOV2, and TAOS2). It is now well established that ANO1 is an eight-transmembrane protein and that ANO1 functions as a calcium-activated chloride channel (CaCC) localized at the plasma membrane (8–10). ANO1 is physiologically expressed in multiple tissues, including secretory epithelia, smooth muscles, and sensory neurons, and is involved in regulation of airway fluid secretion, gut motility, secretory functions of exocrine glands, vascular smooth muscle contraction, and nociception (8–12). Therefore, dysregulation of ANO1 plays a critical role in several disease states, including pulmonary diseases, hypertension, diarrhea, and cancer (2–7, 11, 13). Not surprisingly, knockout of ANO1 is embryonic lethal (14). Amplification of ANO1 correlates with poor overall survival in HNSCC and breast cancer (6, 15). Down-regulation of ANO1 protein levels by RNAi in HNSCC, ESCC, prostate cancer, and breast cancer cells inhibits proliferation *in vitro* and *in vivo* by inhibiting activation of epidermal growth factor receptor and MAPK/AKT-signaling pathways and thus establishes ANO1 as an important survival factor in these cells (6, 7, 15). Furthermore, ANO1 has been implicated to regulate tumor cell motility and metastasis via interaction with the cytoskeletal proteins of the ezrin/radixin/moesin family (16). Therefore, ANO1 may represent a promising target for cancer therapy.

The identification of inhibitors targeting the CaCC activity of ANO1 suggests that biochemical inhibition of ANO1 function is feasible and that targeted modulation could be of therapeutic benefit. However, many of the reported ANO1 inhibitors are natural products with a broad specificity and show activity only in the high micromolar range (17). Screens have identified more potent and more specific inhibitors of ANO1 (17–20). CaCC_inh_-A01 has been shown to inhibit ANO1-dependent chloride conductance in cells and to decrease proliferation of ANO1-dependent cell lines (6, 17). More recently, T16A_inh_-A01, another small molecule inhibitor of ANO1 biochemical activity, has been reported (18). However, direct binding of neither T16A_inh_-A01 nor CaCC_inh_-A01 to ANO1 has been shown, and the mechanism of inhibition is still unknown. It remains unclear how inhibition of a transient chloride current results in inhibition of proliferation over a multiple-day time course.

We sought to explore the mechanism of CaCC_inh_-A01-dependent inhibition of cell proliferation using a combined experimental and *in silico* approach. We show that CaCC_inh_-A01 but not T16A_inh_-A01 inhibits proliferation of ANO1-amplified cell lines, indicating that inhibition of ANO1 biochemical activity was not sufficient to decrease cell proliferation because both inhibitors inhibited the channel activity of ANO1. Rather we show that CaCC_inh_-A01 inhibits cell proliferation by promoting endoplasmic reticulum (ER)-associated proteasomal degradation of ANO1. By performing structure-activity analysis based on the x-ray crystal structure of CaCC_inh_-A01, we developed a pharmacophore model and identified new inhibitors of ANO1-dependent cell proliferation, all of which facilitated ANO1 degradation. Our data demonstrate that inhibition of ANO1 activity is not sufficient to inhibit proliferation of ANO1-amplified cell lines, indicating that both ANO1 channel activity and ANO1 protein are required for its role in cancer. Furthermore, we have shown the utility of *in silico* pharmacophore modeling methods to guide the understanding of biological phenomena involving proteins that have only limited structural information available and are not readily accessible via experimentation.

## EXPERIMENTAL PROCEDURES

### 

#### 

##### In Silico

Virtual screens and pharmacophore alignments were performed with the Cresset suite of programs, including FieldAlign and FieldScreen (21, 22). Structure-activity relationship and quantitative structure-activity relationship analysis were performed using Schrödinger's chemoinformatics program, Canvas (Canvas, version 1.5, Schrödinger, LLC, New York) (23, 24). Surface area and volume calculations were computed within Schrödinger's Maestro (Maestro, version 9.3, Schrödinger).

##### Quantitative Structure-Activity Relationship

The 25 most potent compounds were considered. The data were split randomly into 75% training and 25% testing subsets. The most robust model proved to be the two-dimensional multiple linear regression model. *R*^2^ for the training set and the *Q*^2^ for the test set were both about 0.75, and the S.E. in the modeled value of the hit score was 0.06–0.07. Cell culture was performed as described previously (6).

##### Compounds

T16A_inh_-A01 (Tocris), digallic acid (Toronto Research Chemicals), pentagalloyl glucose (PGG) (Sigma-Aldrich), tannic acid (Bosche Scientific), CaCC_inh_-NV2 and -NV7, brefeldin A (BFA), chloroquine, MG132 (Sigma-Aldrich), cycloheximide (Calbiochem), CaCC_inh_-A01 (Specs), CaCC_inh_-NV4 (ChemDiv), and all other compounds obtained from our internal archive were dissolved in DMSO to a final concentration of 10 mm. If not noted otherwise, cells were treated with 10 μm compound.

##### Crystal Structure Determination and Refinement of CaCC_inh_-A01

The Ca^2+^ complex of CaCC_inh_-A01 was prepared by adding 10 μl of a saturated solution of Ca(OH)_2_ in MeOH to a solution of 1 mg of CaCC_inh_-A01 in 200 μl of EtOH and letting the solvents evaporate at ambient temperature. Colorless crystals were obtained within 1 day. Diffraction data were collected with a Bruker AXS SMART 6000 CCD detector on a three-circle platform goniometer with Cu(Kα) radiation (λ = 1.54178 Å) from a Microstar rotating anode generator equipped with Incoatec multilayer mirrors. A semiempirical absorption correction was applied, based on the intensities of symmetry-related reflections measured at different angular settings (maximum and minimum transmission 0.8494 and 0.7290). The structure was solved by dual-space recycling methods and refined on F2 with the SHELXTL suite of programs.

CaCC_inh_-A01 crystallizes in the triclinic space group P-1 as a hexamer with 0.33 eq of ethanol. The (disordered) ethanol molecule is coordinating Ca25. The disorder was modeled by refining two different orientations (*a*, *b*) with a 0.55 (*a*) to 0.45 (*b*) distribution. Two residual electron density peaks close to the inversion center were interpreted as half a methanol molecule. Although the respective hydrogen atoms at C56 and O57 were calculated in idealized positions, they interfere with H2 from one of the furane moieties. This might indicate that the residual electron density could also be interpreted as partially occupied water sites. All three CaCC_inh_-A01 ligands are disordered in the region of the *tert*-butyl group. This disorder was modeled by refining two different orientations (*a*, *b*) with the following distributions (*a*:*b*): 0.73:0.27 in ligand 1, 0.62:0.38 in ligand 2, and 0.64:0.36 in ligand 3. Bond lengths, angles, and displacement parameters of all minor occupancy orientations were restrained to be similar to those of the major occupancy orientations. For the major occupancy orientation atoms, anisotropic displacement parameters were used, and for the minor occupancy orientation atoms, isotropic displacement parameters were used (except for the ethanol molecule). Hydrogen atoms were calculated in idealized positions and refined using a riding model. Final data: C_113_ H_144_ N_6_ O_31_ S_6_ Ca_3;_ *M*_r_ = 2394.94 (calculated for hexamer of CaCC_inh_-A01 with 3 Ca^2+^, 2 ethanol, 1 methanol), crystal size 0.12·0.08·0.06 mm^3^ (grown from methanol/ethanol), triclinic, space group *P* −1 (No. 2) with *a* = 14.329(4), *b* = 15.048(4), *c* = 15.612(4) Å, α = 69.862(14)°, β = 72.486(13)°, γ = 79.705(12)°, *V* = 3003.4(14) Å^3^, *Z* = 1, *D_c_* = 1.324 g·cm^−3^, μ = 2.811 mm^−1^, *F*(000) = 1268, 64,238 reflections measured, 10,825 independent, *R*_int_ = 0.0392, 3.12° < θ < 68.33°, *T* = 100(2) K, 832 parameters, 687 restraints, *R*_1_ = 0.0395, *wR*_2_ = 0.1062 for 9408 reflections with *I* > 2σ(*I*), R_1_ = 0.0472, *wR*_2_ = 0.1145 for all 10,825 data, GoF = 1.030, restrained GoF = 1.038, res. el.dens. = +0.59/−0.36 e·Å^3^.

Copy number analysis and quantitative PCR were performed as described previously (6). Cell viability and colony formation assays were performed as described (6). Cell viability was determined after a 72-h treatment with compound. Colonies were stained after 10–14 days. Compounds were rank-ordered by hit score.

The hit score is given by Equation 1,

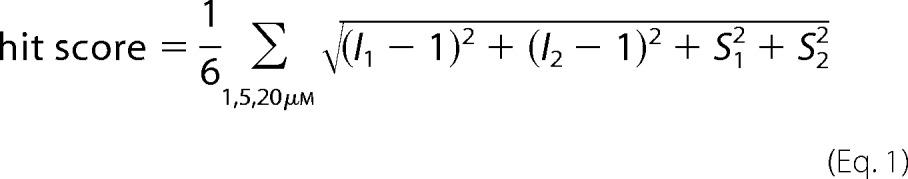
 where *I* and *S* are the average relative cell viability (normalized to DMSO-only-treated cells), with *I*_1_ and *I*_2_ as the two ANO1 inhibition-insensitive cell lines (Te1 and KYSE150) and *S*_1_ and *S*_2_ as the two CaCC_inh_-A01-sensitive and ANO1-dependent cell lines (Te11 and FaDu). Because the assays were run at varying concentrations of inhibitor (1, 5, and 20 μm), the summation is taken over the results obtained at all three concentrations. CaCC_inh_-A01 resulted in a hit score of 0.40. Compounds with a hit score smaller than 0.40 + 10% (hit score <0.44) were classified as a “hit” in subsequent assays.

Generation of stable cell lines expressing shRNAs was performed as described (6, 25, 26). Native PAGE was performed as described (25, 26). Cross-linking was performed as described (27).

##### Immunoprecipitation

Cells were lysed in radioimmune precipitation buffer (Cell Signaling), and ∼500 μg of total protein was incubated with an anti-ubiquitin antibody (Millipore, FK2) for 1 h at 4 °C. Antibodies were precipitated using Protein G-coated Dynabeads (Invitrogen), and bound protein was eluted in Laemmli buffer (Invitrogen) for 10 min at 70 °C. Western blotting was performed as described (6).

##### Immunofluorescence

Cells were fixed in 4% paraformaldehyde in PBS for 20 min at room temperature; incubated in 0.1% SDS/PBS for 5 min; blocked in 5% BSA, PBS, 0.3% Triton X-100 for 1 h at room temperature; and incubated in primary antibody (anti-ANO1, SP31, Abcam, 1:200; anti-β-catenin, Cell Signaling, 1:500) overnight in 3% BSA, PBS, 0.3% Triton X-100. Binding of the primary antibody was visualized by Alexa 594- or 488-labeled secondary antibodies (1:3000; Invitrogen). Coverslips were mounted, and nuclei were stained with DAPI (Vectashield, Vector Labs). Images were taken with an Olympus IX-81 inverted microscope (Olympus). Cell cycle analysis was performed as described previously (6).

##### QPatch

Whole-cell currents were measured using planar (QPatch, Sophion) patch clamp electrophysiology (28), as described (6).

## RESULTS

### 

#### 

##### Inhibition of ANO1 Activity Is Not Sufficient to Inhibit ANO1-dependent Cell Proliferation

**FIGURE 1.**
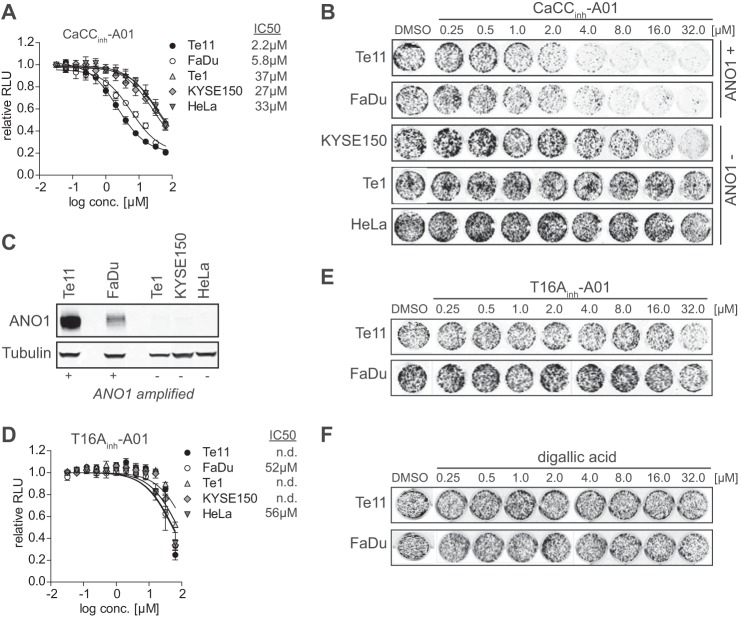
**Inhibition of ANO1-dependent chloride conductance is not sufficient to inhibit ANO1-dependent cell proliferation.** *A*, effect of CaCC_inh_-A01 on proliferation of the indicated cell lines (mean ± S. E. (*error bars*), *n* > 3). Cell viability was determined after 72 h of treatment with the indicated compounds or solvent. *B*, effect of CaCC_inh_-A01 on colony formation of the indicated cell lines. Cells were seeded at low density and treated with the indicated compounds or solvent for 10–14 days. Colonies were stained using crystal violet. Representative images of stained colonies in a 24-well plate are shown. *C*, ANO1 expression was examined in the indicated cell lines by Western blotting. Representative blots are shown. *D*, effect of T16A_inh_-A01 on proliferation of the indicated cell lines (mean ± S.E., *n* > 3) analyzed as in *A. E* and *F*, effect of T16A_inh_-A01 (*E*) or digallic acid (*F*) on colony formation of the indicated cell lines, analyzed as in *B*. RLU, relative light units.

ANO1 is amplified and highly expressed in HNSCC, ESCC, and breast cancer (6, 7, 15). ANO1 is an important oncogenic survival factor in these cancers, and RNAi-mediated knockdown of ANO1 in Te11 (HNSCC) and FaDu (ESCC) cells diminished cell proliferation (6). Treatment of ANO1-amplified Te11 and FaDu cells with the ANO1 inhibitor CaCC_inh_-A01 decreased colony formation and cell viability over a >72-h time course. Consistent with ANO1 being the primary target of this compound, cells without ANO1 amplification and low expression of ANO1 (e.g. KYSE150, Te1, and HeLa) were found to be insensitive to CaCC_inh_-A01 (Fig. 1, *A–C*, and Table 1). Interestingly, T16A_inh_-A01 and digallic acid did not inhibit the proliferation of ANO1-dependent cell lines, although both compounds have been reported to inhibit ANO1 activity (Fig. 1, *E* and *F*) (18). These data suggest that inhibition of ANO1 activity alone is not sufficient to inhibit ANO1-dependent cell proliferation.

**TABLE 1:** **IC_50_ values for inhibition of cell viability by CaCC_inh_-A01 in correlation with ANO1 amplification status** Data represent the mean of three independent experiments. NA, non-amplified.

Cell line	Copy no. ANO1	IC_50_ CaCC_inh_-A01
		μ*m*
FaDu	14	8.5
KYSE140	4	8.0
KYSE510	6	3.1
Te11	9	2.9
Te6	9	2.5
Hek293	NA	>25
HeLa	NA	>25
KYSE150	NA	>25
KYSE450	NA	16
KYSE70	NA	>25
Te1	NA	16
Te9	NA	17

##### Small Molecule Crystal Structure of CaCC_inh_-A01 Shows Calcium Binding

**FIGURE 2.**
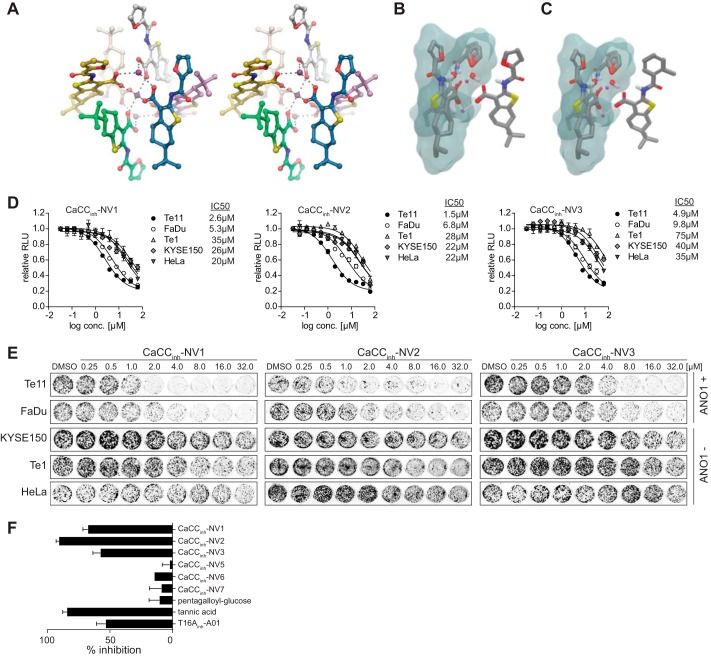
**Virtual screening identifies novel inhibitors of ANO1-dependent cell proliferation.** *A*, *cross-eyed stereoimage* of the crystal structure of CaCC_inh_-A01. No hydrogen atoms have been added, and water and solvent molecules have been omitted for clarity. Atoms are *colored* by element, with *dark purple*, *red*, *blue*, and *yellow* representing calcium, oxygen, nitrogen, and sulfur, respectively. The carbons are *colored* by molecule for clarity. *B* and *C*, crystal structure unit cell of CaCC_inh_-A01 used to model CaCC_inh_-A01 analogs. The excluded volume used from the crystal structure unit cell is shown *outlined* in *blue*, whereas the modeled compounds (*B*, CaCC_inh_-A01; *C*, CaCC_inh_-NV2) are shown on the *right*. The calcium ions are shown in *purple. D* and *E*, effect of CaCC_inh_-NV1–3 on cell viability (*D*) and colony formation (*E*) of the indicated cell lines (mean ± S.E. (*error bars*), *n* = 4; representative images of stained colonies in a 24-well plate are shown). *F*, relative inhibition of ANO1 currents in Te11 cells as compared with CaCC_inh_-A01. The indicated compounds were tested at 30 μm (mean ± S.E., *n* = 3).

To understand the mechanism of CaCC_inh_-A01 activity, we performed structure-activity analysis around CaCC_inh_-A01. We set out to explore the presence of a carboxylic acid on CaCC_inh_-A01 because this functional group is a putative chelator of metal ions, including calcium. We speculated that chelation of calcium may play a role in the activity of CaCC_inh_-A01 because ANO1 is a calcium-activated chloride channel. In order to explore this hypothesis, we asked what form CaCC_inh_-A01 would take when crystallized in the presence of calcium. An in-house x-ray crystal structure shows a hexameric unit cell of CaCC_inh_-A01 in the P-1 space group chelating three calcium atoms (Fig. 2*A*). Within the structure, it is evident that there is a symmetric, intercalated CaCC_inh_-A01 trimer architecture centered on a central calcium atom inversion center, suggesting that chelation of calcium may be important for the activity of CaCC_inh_-A01. CaCC_inh_-A01 is known to not affect intracellular calcium levels (17); however, because ANO1 is regulated by multiple calcium-dependent mechanisms (29), we speculated whether the ability of the compound to chelate local stores of calcium was sufficient to exhibit an effect on ANO1-dependent cell proliferation. We tested 70 known calcium chelators for an effect on cell proliferation in two ANO1-dependent (Te11, FaDu) and two ANO1-independent (KYSE150, Te1) cell lines. None of the calcium chelators tested inhibited ANO1-dependent cell proliferation, suggesting that calcium chelation alone is not sufficient to account for the activity of CaCC_inh_-A01 in ANO1-amplified cell lines (data not shown). In addition, we have tested the effect of EGTA-AM (a cell membrane-permeable form of EGTA) for an effect on the proliferation of Te11, FaDu, KYSE150, and Te1 cells. EGTA-AM inhibited proliferation of all four cell lines with an IC_50_ of ∼10 μm, indicating that it acts via an ANO1-independent mechanism. This further corroborates our finding that the calcium chelation functionality of CaCC_inh_-A01 is important but not sufficient for the compound's activity on proliferation seen in ANO1-amplified cell lines.

##### Chelating Functionality Is Necessary for CaCC_inh_-A01 Activity

We speculated that calcium chelation and a specific binding interaction of CaCC_inh_-A01 with ANO1 together drive potency. Accordingly, we sought to identify additional compounds that shared those pharmacophoric features of CaCC_inh_-A01. An *in silico* screen was performed, where a CaCC_inh_-A01 crystallographic monomer was used as a template (Fig. 2, *B* and *C*). A total of 170 compounds with pharmacophore features similar to those of CaCC_inh_-A01 were identified and tested for their effect on ANO1-dependent cell proliferation (data of most the potent inhibitors are shown in Table 2, CaCC_inh_-NV1–3). The compounds identified in this screen represented numerous potential chelators, including carboxylates, nitriles, amides, and esters, yet only the carboxylate-containing molecules showed activity in ANO1-dependent cell proliferation assays. CaCC_inh_-NV1, -NV2, and -NV3 decreased the proliferation of ANO1-dependent cell lines in a concentration-dependent manner and inhibited ANO1-dependent current in Te11 cells (Fig. 2, *D–F*).

**TABLE 2:**
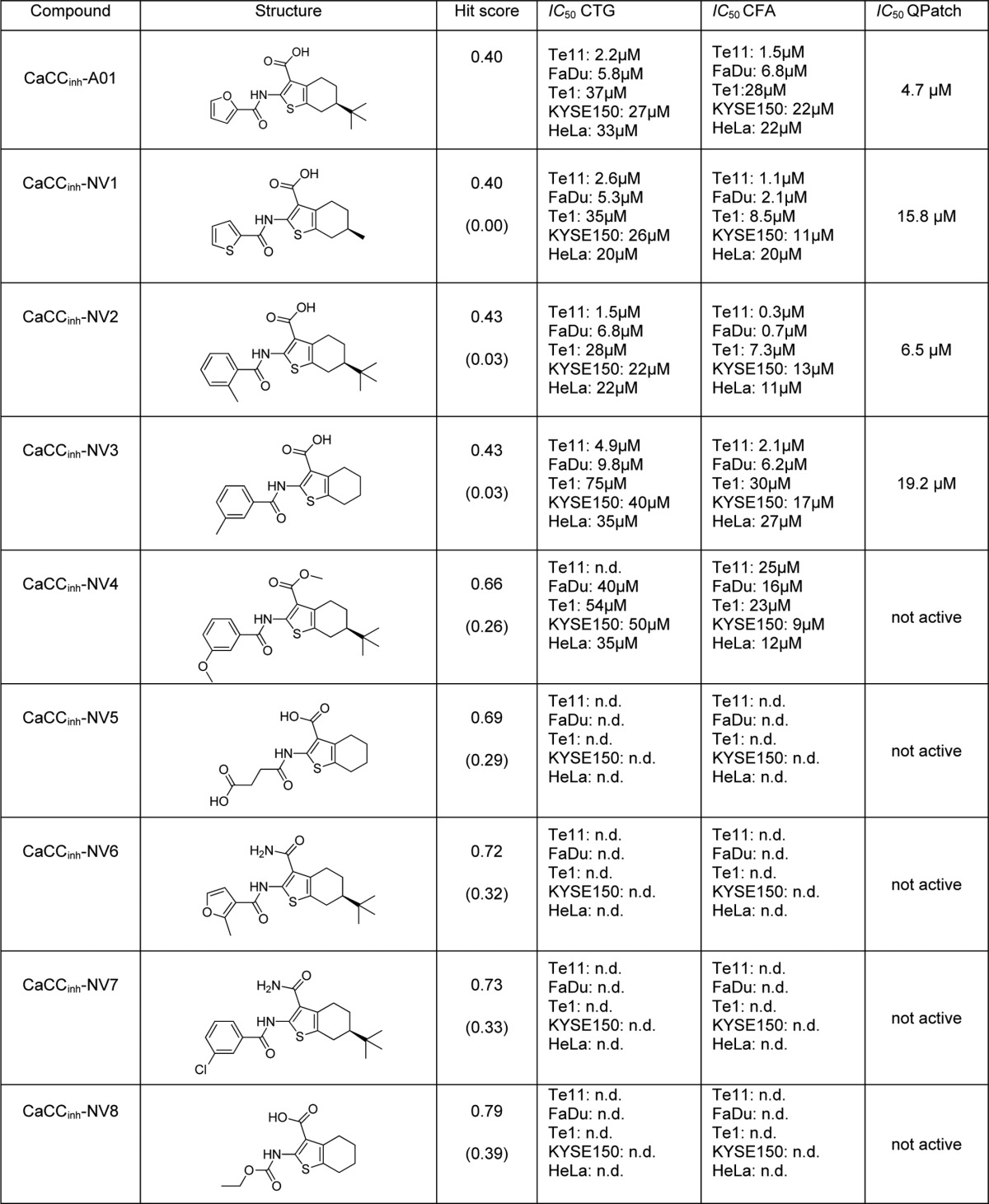
**Summary of the structural and biochemical characteristics for all newly identified molecules** Values of the hit score relative to that of CaCC_inh_-A01 (in parentheses) and IC_50_ are given.

To investigate whether the presence of a chelating group is necessary for a compound's potency, we performed an *in silico* screen using the CaCC_inh_-A01 crystal structure, where the carboxylate was replaced by hydrogen before being used as the template, and tested the predicted compounds in our proliferation assay. None of the 64 compounds identified from this screen showed activity in our cellular assay despite their high degree of similarity to the template molecule (examples shown in Table 2, CaCC_inh_-NV4–8). This supports the hypothesis that both the hydrophobic interaction (hydrophobic bulk, missing in CaCC_inh_-NV5 and -NV8) and calcium chelation moiety (carboxylic acid, missing in CaCC_inh_-NV4, -NV6, and -NV7) present in CaCC_inh_-A01 are necessary for potency in cells. This is corroborated via *in silico* analysis. We developed a quantitative structure-activity relationship model to describe our hit score: hit_score = −0.34*acidic_hydrogens + 0.019*AlogP. Notably, the developed quantitative structure-activity relationship model employs only two descriptors, which count the number of carboxylic acids and describe hydrophobicity, illustrating the importance of these two attributes to the hit score. Activity cliff analysis further supported this finding. Thus, we describe the first pharmacophore model for inhibitors of ANO1-dependent cell proliferation.

##### CaCC_inh_-A01 Pharmacophore Model Can Be Used to Identify Inhibitors of ANO1-dependent Cell Proliferation

**FIGURE 3.**
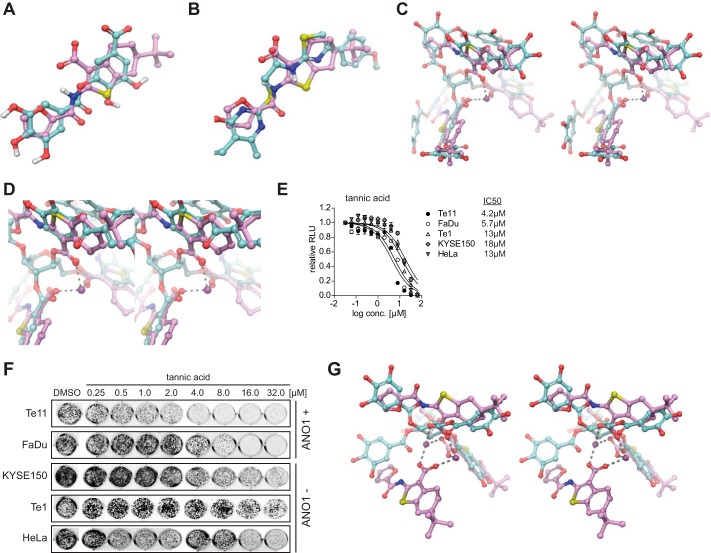
**Tannic acid resembles CaCC_inh_-A01 trimer, promotes degradation of ANO1, and inhibits ANO1-dependent cell proliferation.** *A*, flexible ligand alignment of digallic acid with CaCC_inh_-A01. Atoms are *colored* by element, with *white*, *red*, *blue*, and *yellow* representing hydrogen, oxygen, nitrogen, and sulfur, respectively. The carbons are *colored* by molecule for clarity, where *turquoise* and *lilac* correspond to digallic acid and CaCC_inh_-A01, respectively. Note that only polar hydrogens are displayed for clarity. *B*, flexible ligand alignment of T16A_inh_-A01 with CaCC_inh_-A01. Atoms are *colored* by element with *red*, *blue*, and *yellow* representing oxygen, nitrogen, and sulfur, respectively. The carbons are *colored* by molecule for clarity, where *turquoise* and *lilac* correspond to T16A_inh_-A01 and CaCC_inh_-A01, respectively. Note that only polar hydrogens are displayed for clarity. *C*, *cross-eyed stereoimage* of flexible ligand alignment of tannic acid with the CaCC_inh_-A01 crystallographic trimer. Atoms are *colored* as described in the legend to Fig. 2. The carbons are *colored* by molecule for clarity, where *turquoise* and *lilac* correspond to tannic acid and the CaCC_inh_-A01 crystallographic trimer, respectively. One calcium ion of the CaCC_inh_-A01 crystal structure is shown as a *purple sphere. D*, *close-up cross-eyed stereoimage* of flexible ligand alignment of tannic acid with the CaCC_inh_-A01 crystallographic trimer. *E* and *F*, effects of tannic acid on cell proliferation (*E*) and colony formation (*F*) of the indicated cell lines (mean ± S.E. (*error bars*), *n* = 4; representative images of stained colonies in a 24-well plate are shown). *G*, *cross-eyed stereoimage* of flexible ligand alignment of pentagalloyl glucose with the CaCC_inh_-A01 crystallographic trimer. Atoms are *colored* by element, with *dark purple*, *red*, *blue*, and *yellow* representing calcium, oxygen, nitrogen, and sulfur, respectively. The carbons are *colored* by molecule for clarity, where *turquoise* and *lilac* correspond to pentagalloyl glucose and the CaCC_inh_-A01 crystallographic trimer, respectively. Note that no hydrogens are displayed for clarity.

We used our pharmacophore model to rationalize the observed differential activity of T16A_inh_-A01, digallic acid, and CaCC_inh_-A01. Although all of these molecules inhibit the CaCC activity of ANO1 (18, 30), only CaCC_inh_-A01 had an inhibitory effect on ANO1-dependent cell proliferation in tumor cells. T16A_inh_-A01 lacks a carboxylic acid, a critical feature of the pharmacophore model (Fig. 3*A*). Digallic acid, although containing a carboxylic acid, lacks much of the hydrophobic bulk of CaCC_inh_-A01 (Fig. 3*B*). Thus, both T16A_inh_-A01 and digallic acid significantly deviate from our pharmacophore model. Given that digallic acid deviates from the pharmacophore by lacking hydrophobic bulk, we hypothesized that tannic acid, being a glucose-linked pentamer of digallic acid with reported inhibitory activity on ANO1 (30), would fulfill this requirement and inhibit ANO1-dependent cell proliferation. In the alignment of tannic acid with the trimeric subunit of the hexameric crystal structure of CaCC_inh_-A01, henceforth referred to as “trimer,” ∼1.5 units of digallic acid overlap with each unit of CaCC_inh_-A01 (Fig. 3, *C* and *D*). Thus, in tannic acid, the lack of bulk in the individual digallic acid units is compensated for by a larger numbers of these units. Furthermore, the glucoso-ester moieties of tannic acid align with the carboxylates of the CaCC_inh_-A01 trimer, theoretically allowing for chelation of a calcium cation, potentially stabilizing the conformation. Therefore, tannic acid lies within the confines of our pharmacophore model. In agreement with our hypothesis, tannic acid indeed inhibited cell proliferation of ANO1-dependent cell lines (Fig. 3, *E* and *F*).

We tested the potency of another tannic acid analog shown to inhibit ANO1-dependent chloride currents, PGG (30). PGG does not align with the trimer of CaCC_inh_-A01 (Fig. 3*G*) and did not show a specific effect on the proliferation of ANO1-dependent cells and showed a nonspecific inhibition of proliferation in all tested cell lines (data not shown). PGG is known to inhibit cell proliferation in tumor types that do not express ANO1, so its cytotoxic effect in tumor cells is not due to ANO1 inhibition (31). Thus, we posit that the pharmacophore model for inhibitors also includes conglomeration of small molecules into a multimeric structure that resembles tannic acid, explaining the observed similarity in activity of these seemingly disparate molecules. The characteristics necessary for ANO1 inhibitors to exhibit an effect on ANO1-dependent cell proliferation can be described by our pharmacophore model.

##### CaCC_inh_-A01 Decreases ANO1 Protein Levels

**FIGURE 4.**
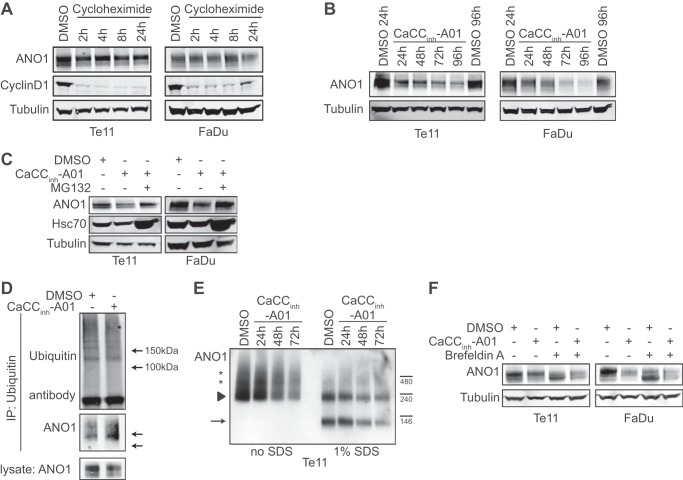
**CaCC_inh_-A01 promotes the endoplasmic reticulum-associated, proteasomal degradation of ANO1.** *A*, analysis of ANO1 protein stability. Cells were treated for the indicated times with 50 μm cycloheximide, and ANO1 protein levels were analyzed by Western blotting. Cyclin D1 was used as a control for the activity of cycloheximide. Representative blots are shown. *B*, effects of CaCC_inh_-A01 on ANO1 protein levels were examined by Western blotting after the indicated time of treatment with 10 μm CaCC_inh_-A01 or DMSO. Tubulin was used as a loading control. Representative Western blots are shown. *C*, effects of MG132 on CaCC_inh_-A01-induced degradation of ANO1. Cells were pretreated with 10 μm CaCC_inh_-A01 for 6 h before the co-treatment with 20 μm MG132 for an additional 18 h. Representative Western blots are shown. Hsc70 was used to control the effect of MG132. *D*, immunoprecipitation (*IP*) of mono- and polyubiquitinated proteins in Te11 cells treated with 10 μm CaCC_inh_-A01 or DMSO using an anti-ubiquitin antibody. Note the increase in the molecular weight for ANO1 due to ubiquitination. Representative Western blots are shown. *E*, representative Western blot of a blue native polyacrylamide gel is shown. Te11 cells were treated as indicated with 10 μm CaCC_inh_-A01 or DMSO. 1% SDS was added to the samples (*lanes 6–9*) prior loading to the gel. *Arrow*, expected size of ANO1 monomer; *arrowhead*, expected size of ANO1 dimer; *stars*, ANO1 multimers or complexes. *F*, effects of 10 μm brefeldin A on CaCC_inh_-A01-induced degradation of ANO1. Representative Western blots are shown.

We then investigated the cellular mechanism underlying the inhibitory effect of CaCC_inh_-A01 on proliferation of ANO1-expressing cells. Given that genetic knockdown of ANO1 decreases cell viability in Te11 and FaDu cells (6), we asked whether the binding of CaCC_inh_-A01 to ANO1 might affect the expression or stability of the protein in addition to inhibiting its channel activity. Because the stability of ANO1 in cells has not been studied previously, we investigated the protein half-life of ANO1 in cells. Our results show that the half-life of ANO1 protein is greater than 24 h (Fig. 4*A*). In order to test for an effect of CaCC_inh_-A01 on ANO1 expression or stability, Te11 and FaDu cells were treated with CaCC_inh_-A01. ANO1 mRNA and protein levels were monitored over a time course by quantitative PCR and Western blotting, respectively. Treatment of Te11 and FaDu cells with CaCC_inh_-A01 had no effect on ANO1 mRNA levels (data not shown) but led to a decrease in ANO1 protein levels in a time-dependent manner, indicating post-transcriptional regulation of ANO1 protein levels after CaCC_inh_-A01 treatment (Fig. 4*B*).

##### CaCC_inh_-A01 Promotes Proteasomal Degradation of ANO1

Given the long half-life of ANO1, we speculated that CaCC_inh_-A01 might affect ANO1 protein levels by inducing degradation of the protein via the lysosome- or proteasome-dependent degradation pathway. Whereas inhibition of lysosomal degradation using chloroquine had no effect on ANO1 protein levels in Te11 or FaDu cells (data not shown), inhibition of the proteasome with MG132 prevented ANO1 degradation after CaCC_inh_-A01 treatment in both cell types (Fig. 4*C*). Consistent with this result, we found that CaCC_inh_-A01 treatment increased the ubiquitination of ANO1 (Fig. 4*D*). Taken together, these data provide evidence that CaCC_inh_-A01 promotes proteasome-dependent degradation of ANO1.

##### CaCC_inh_-A01 Does Not Affect the Dimerization of ANO1

We then investigated the mechanism by which CaCC_inh_-A01 induces proteasome-dependent degradation of ANO1. We hypothesized that CaCC_inh_-A01 may increase degradation of ANO1 by affecting its stability (*e.g.* by disrupting critical protein-protein contacts), compromising the structure of the protein, and leaving the protein vulnerable to degradation. ANO1 has been shown to form dimers when overexpressed in HEK293 cells (25, 26, 32), and we hypothesized that CaCC_inh_-A01 might interfere with the dimerization of ANO1. To explore this hypothesis, we tested the effects of CaCC_inh_-A01 on the stability of the dimeric form of ANO1 and on the interaction of ANO1 with other proteins by performing non-denaturing blue native polyacrylamide gel electrophoresis followed by Western blot analysis for ANO1. Under non-denaturing conditions, an ANO1 antibody stained for several bands (Fig. 4*E*), including the main signal observed around 240 kDa, representing dimeric ANO1. Several bands of higher molecular weight were detectable in lysates of Te11 cells, indicating the existence of oligomers or complexes of ANO1 with other proteins. Partial denaturation of the natively extracted ANO1 by the addition of 1% SDS to the sample resulted in a decrease of the 240 kDa band and disappearance of higher order complexes. A faster migrating band around 120 kDa was detected, representing the monomeric form of ANO1. Treatment of Te11 cells with CaCC_inh_-A01 decreased total ANO1 protein levels but did not affect the quaternary structure of total ANO1 protein in the cells. Similar results were obtained in FaDu cells (data not shown). Consistently, treatment of Te11 and FaDu cells with CaCC_inh_-A01 had no effect on the ratio between the monomeric and dimeric form of ANO1 on the cell membrane after cross-linking with the cell-impermeable cross-linker BS3 but consistently decreased total ANO1 protein levels (data not shown). These results provide evidence that ANO1 forms dimeric and multimeric complexes under endogenous expression in tumor cells and that the formation of these complexes is not affected by CaCC_inh_-A01.

##### CaCC_inh_-A01 Facilitates ER-associated, Proteasomal Turnover of ANO1

Next, we tested whether CaCC_inh_-A01 treatment induces the internalization from the plasma membrane and the transfer of ubiquitinated ANO1 to the proteasome. Immunofluorescence staining of ANO1 after treatment of Te11 and FaDu cells with CaCC_inh_-A01 did not reveal any vesicular staining of ANO1 and rather caused a gradual overall decrease of total ANO1 staining intensity in the cells (data not shown). This observation led us to hypothesize that CaCC_inh_-A01 interferes with the turnover of ANO1 protein by facilitating the degradation of the intracellular pool of ANO1. To test this hypothesis, we treated Te11 and FaDu cells with BFA (33, 34), which led to a shift in the molecular weight for ANO1, representing the partially glycosylated, non-(Golgi)maturated form of ANO1 and indicating that ANO1 undergoes intracellular recycling by retrograde transport (Fig. 4*F*). To test whether the CaCC_inh_-A01-dependent turnover of ANO1 occurs in the ER or a post-Golgi compartment, we treated the cells with a combination of BFA and CaCC_inh_-A01. Treatment of Te11 and FaDu cells with CaCC_inh_-A01 in the presence of BFA led to a decrease in ANO1 protein levels as compared with BFA treatment alone (compare *lanes 3* and *4* and *lanes 7* and *8*). These data demonstrate that degradation of ANO1 still appears in the presence of BFA, indicating that the degradation occurs in a pre-Golgi compartment. Taken together, these results suggest that CaCC_inh_-A01 interferes with the intracellular turnover of ANO1 by promoting the ER-associated, proteasomal degradation of ANO1.

##### CaCC_inh_-A01 Inhibits Cell Proliferation by Reducing ANO1 Protein Levels

**FIGURE 5.**
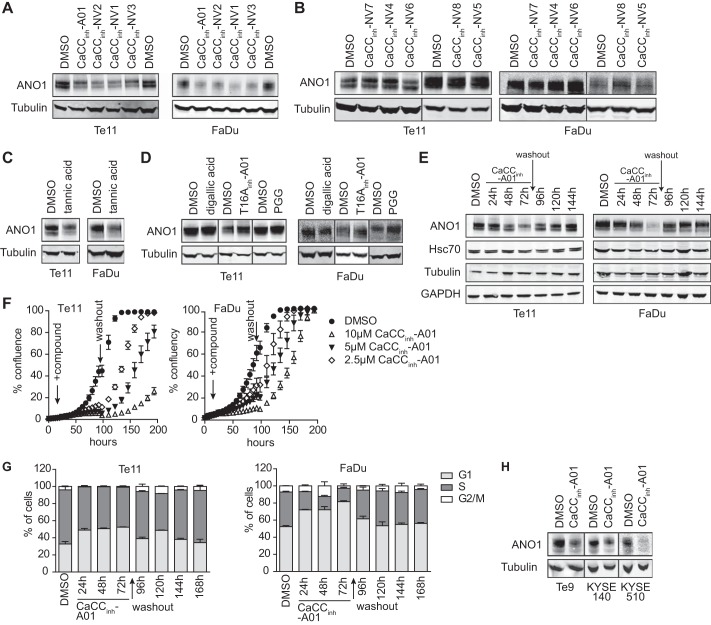
**CaCC_inh_-A01 inhibits proliferation by promoting degradation of ANO1.** *A*, effects of CaCC_inh_-NV1–3 on ANO1 protein levels were examined by Western blotting after 72 h of treatment with 10 μm compound or DMSO. Representative Western blots are shown. *B*, effects of CaCC_inh_-NV4–8 on ANO1 protein levels examined as in *A. C* and *D*, effects of tannic acid, digallic acid, T16A_inh_-A01, and PGG on ANO1 protein levels examined as in *A. E*, effect of CaCC_inh_-A01 washout on ANO1 protein levels assessed as described in *A. F*, Te11 and FaDu cell proliferation in the presence and after washout of the indicated concentrations of CaCC_inh_-A01 or DMSO was monitored using the Incucyte system (mean ± S.E. (*error bars*), *n* = 4). *G*, cell cycle analysis of Te11 and FaDu cells in the presence of and after washout of the indicated concentrations of CaCC_inh_-A01 or DMSO. Cells were stained with propidium iodide, and DNA content was analyzed by FACS (mean ± S.E., *n* = 3). *H*, effects of CaCC_inh_-A01 on ANO1 protein levels were examined in the indicated cell lines by Western blotting after 72 h of treatment with 10 μm CaCC_inh_-A01 or DMSO. Representative Western blots are shown.

Given that RNAi-mediated knockdown of ANO1 decreases cell viability in Te11 and FaDu (6), we hypothesized that the inhibitory effect of CaCC_inh_-A01 on cell proliferation results from compound-induced reduction in ANO1 protein levels. Consistent with this, all of our newly identified inhibitors of ANO1-dependent cell proliferation (CaCC_inh_-NV1, CaCC_inh_-NV2, and CaCC_inh_-NV3) as well as tannic acid decreased ANO1 protein levels, whereas their structurally similar but inactive counterparts (CaCC_inh_-NV4, CaCC_inh_-NV5, CaCC_inh_-NV6, CaCC_inh_-NV7, and CaCC_inh_-NV8) and pentagalloyl glucose did not (Fig. 5, *A–D*). Intriguingly, treatment with T16A_inh_-A01 or digallic acid did not affect ANO1 protein levels, correlating with their lack of effect on ANO1-dependent cell proliferation. This led us to hypothesize that CaCC_inh_-A01 inhibits ANO1-dependent cell proliferation by reducing ANO1 protein levels. Therefore, removal of CaCC_inh_-A01 should lead to recovery of ANO1 protein levels and resume cell proliferation. To test this hypothesis, we treated Te11 and FaDu cells for 72 h with CaCC_inh_-A01, followed by washout of CaCC_inh_-A01 and incubation of the cells in normal growth medium. Samples for cell cycle and Western blot analysis of ANO1 were taken every 24 h, and cell viability was monitored. Treatment of Te11 and FaDu cells with CaCC_inh_-A01 reduced protein levels of ANO1, arrested the cells in the G_1_ phase of the cell cycle, and led to pronounced inhibition of cell growth in both cell lines. Consistent with our hypothesis, washout of CaCC_inh_-A01 completely rescued ANO1 protein levels and restored cell viability (Fig. 5, *E–G*). Notably, CaCC_inh_-A01 also reduced ANO1 protein levels in Te9, KYSE140, and KYSE510 cells (Fig. 5*H*). Taken together, these data provide strong evidence that CaCC_inh_-A01 inhibits proliferation of ANO1-amplified cancer cell lines by decreasing ANO1 protein levels, suggesting that both ANO1-activity and protein are necessary for its role as a survival factor in cancer.

**FIGURE 6.**
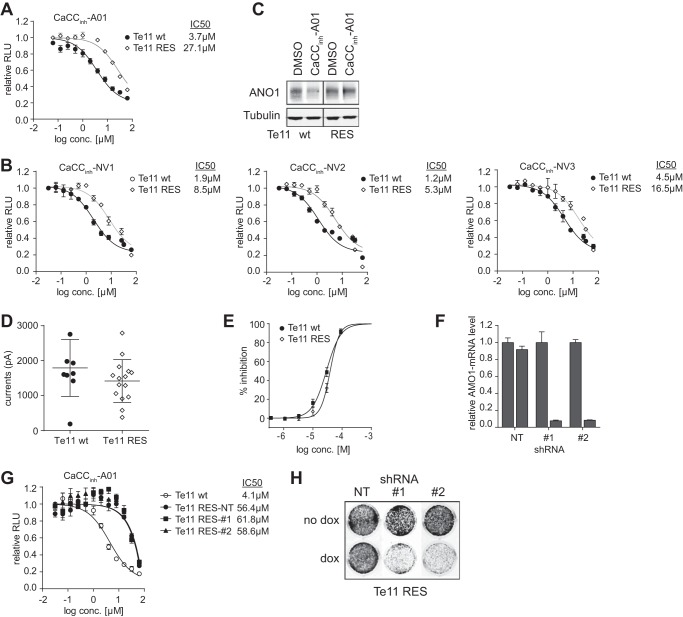
**Resistance to CaCC_inh_-A01-promoted degradation of ANO1 rescues CaCC_inh_-A01-dependent inhibition of cell proliferation.** *A* and *B*, effects of CaCC_inh_-A01 (*A*) and analogs (*B*) on proliferation of parental Te11 cells and CaCC_inh_-A01-resistant Te11 cell pools (mean ± S.E. (*error bars*), *n* = 4, 72-h treatment). *C*, effects of CaCC_inh_-A01 on ANO1 protein levels in parental Te11 cells and CaCC_inh_-A01-resistant Te11 cell pools. Representative Western blots are shown. *D*, ANO1-like currents in Te11 WT and RES cells as assessed using QPatch (mean ± S.E., *n* = 10). *E*, percentage of inhibition of ANO1-like currents in Te11 WT and RES cells at the indicated concentrations of CaCC_inh_-A01 (mean ± S.E., *n* = 4). *F*, relative mRNA-level of ANO1 of Te11-RES cells stably expressing doxycycline-inducible shRNA against ANO1 (#*1* and #*2*) or a non-targeting control (*NT*) after 72-h treatment with or without doxycycline as determined by quantitative PCR. Data are normalized to the respective non-doxycycline-treated control and represent the mean ± S.E., *n* = 3. *G*, effect of CaCC_inh_-A01 on proliferation of parental Te11 cells and Te11-RES cells stably expressing shRNA as described in *F* (mean ± S.E., *n* = 3, 72-h treatment). *H*, effect of ANO1 knockdown on colony formation of Te11-RES cells. Te11-RES cells stably expressing doxycycline-inducible shRNA against ANO1 or a non-targeting control were seeded in a 24-well plate colonies in a 24-well plate are shown. *RLU*, relative light units.

To further investigate the dual role of ANO1 in cancer, we generated CaCC_inh_-A01-resistant Te11 cell pools by culturing the cells in 10 μm CaCC_inh_-A01 (∼4-fold IC_50_) for >3 months. Proliferation of the resistant Te11 cell pools was significantly less sensitive to CaCC_inh_-A01 and to all of the newly identified inhibitors of ANO1-dependent cell proliferation (CaCC_inh_-NV1, CaCC_inh_-NV2, and CaCC_inh_-NV3) as compared with parental, wild type Te11 cells (Fig. 6, *A* and *B*) and showed an IC_50_ similar to that of ANO1-negative cells (Fig. 1*A*). There was no difference in sensitivity to ANO1-independent inhibition of proliferation (data not shown). Genomic sequencing of the ANO1 locus did not reveal any acquired mutations in ANO1 in the resistant cell pool (data not shown). ANO1 protein levels in Te11 wild type cells and the CaCC_inh_-A01-resistant cell pool were similar (Fig. 6*C*). QPatch measurements did not reveal a significant difference in ANO1 chloride currents but showed that ANO1-dependent currents were still inhibited by CaCC_inh_-A01 in the resistant cell pool with an IC_50_ similar to that of the parental cells (Fig. 6, *D* and *E*), further supporting our hypothesis that sustained inhibition of ANO1 channel activity is not sufficient to diminish cell proliferation. Furthermore, treatment with CaCC_inh_-A01 decreased ANO1 protein levels in Te11 parental, wild type cells but did not affect ANO1 protein levels in the CaCC_inh_-A01-resistant cell pool (Fig. 6*C*), providing an explanation for the lack of effect on cell proliferation and demonstrating that promotion of ANO1 degradation is responsible for the effect of CaCC_inh_-A01 on proliferation of ANO1-amplified cancer cells. In order to test whether the CaCC_inh_-A01-resistant cells were still dependent on ANO1 or whether the cells had developed an alternative mechanism that made them ANO1-independent and thus resistant to CaCC_inh_-A01, we generated CaCC_inh_-A01 cell lines stably expressing doxycycline-inducible shRNAs against ANO1 (Fig. 6F, #*1* and #*2*) or a non-targeting control (*NT*). Treatment of shRNA#1/#2-expressing cell lines with doxycycline diminished ANO1 expression, whereas doxycycline had no effect on ANO1 expression in the NT control-expressing cell line (Fig. 6*F*). As shown in Fig. 6*G*, the newly generated stable shRNA lines retained resistance to CaCC_inh_-A01. To test the hypothesis that the resistant cells were still dependent on ANO1 expression, we treated the shRNA-expressing Te11-RES cells with doxycycline and measured cell viability in a colony formation assay. Knockdown of ANO1 led to a significant inhibition of cell viability as detected by a decrease in colony formation (Fig. 6*H*). This finding demonstrates that CaCC_inh_-A01-resistant cells still require ANO1 protein for proliferation and is consistent with our model that the observed CaCC_inh_-A01-resistance in Te11-RES can be explained by the lack of degradation of ANO1 rather than the occurrence of alternative, ANO1-independent resistant mechanisms. In summary, our data demonstrate that degradation of ANO1 rather than inhibition of ANO1 channel activity is critical for the inhibition of ANO1-dependent cell proliferation in ANO1-amplified tumor cells.

## DISCUSSION

ANO1 has been identified as an important survival factor in human cancers and contributes to HNSCC, ESCC, and breast cancer tumorigenesis, making it a promising therapeutic target (2–7). CaCC_inh_-A01 has been shown to inhibit ANO1-dependent chloride conductance in cells and to decrease proliferation of ANO1-dependent cell lines (6, 17), but its mechanism of inhibition has remained elusive. In particular, it remains unclear how inhibition of a transient chloride current results in inhibition of proliferation over a multiple-day time course. We explored the mechanism of CaCC_inh_-A01-dependent inhibition of cell proliferation using a combined experimental and *in silico* approach. We show that CaCC_inh_-A01 inhibits ANO1-dependent cell proliferation by reducing ANO1 protein levels in Te11 and FaDu cells. We found that CaCC_inh_-A01 increased the ubiquitination of ANO1 and facilitated ER-associated, proteasomal degradation of ANO1 without affecting its dimerization. CaCC_inh_-A01 interferes with the naturally occurring turnover processes of ANO1, providing an explanation for the observed slow kinetics of CaCC_inh_-A01-induced loss of ANO1 protein. Our results are consistent with the observation that multiple ion channels have been shown to be regulated by endoplasmic reticulum-associated, ubiquitin-dependent degradation (29, 35–39).

Inhibitor-mediated degradation of proteins often originates in hydrophobic interactions of the molecule with protein. These interactions disrupt critical protein contacts, compromising the proteins' structure and eventually leading to degradation (40, 41). The inhibitors described herein and tannic acid contain a marked percentage of hydrophobic surface area. Approximately 21–29% of the surface area is highly hydrophobic (*e.g.* aromatic ring faces). In theory, the faces of the aromatic rings that constitute the hydrophobic surface area could π-stack with residues of ANO1 (*e.g.* tryptophan and tyrosine), ultimately leading to changes in the conformation of ANO1 and eventually degradation. Our model suggests that CaCC_inh_-A01 and the analogs described herein bind to ANO1 and alter the structure and activity of ANO1, thereby facilitating the constitutive, ER-associated, proteasome-dependent turnover of ANO1. However, without a structure for ANO1, locating possible binding sites and validating this hypothesis remains a challenging goal for further studies. ANO1, being an eight-transmembrane helix protein and having no known homologs with solved structures, evades description with homology models. However, our model does not exclude binding of CaCC_inh_-A01 to a second target that regulates ANO1 stability.

We report various inhibitors of ANO1-dependent cell proliferation, identified by structure-activity analysis of the structure of CaCC_inh_-A01, all of which promote degradation of ANO1. A pharmacophore model was developed to describe compounds capable of facilitating ANO1 degradation and inhibiting ANO1-dependent cell proliferation. This model points to the importance of both the carboxylate and hydrophobic bulk as essential features of these compounds. Our model demonstrates that tannic acid promoted degradation of ANO1, whereas the closely related molecule PGG had no selective effect on ANO1-dependent cell proliferation. Importantly, we have shown the utility of *in silico* methods to guide a better understanding of the biological phenomena of the ANO1 channel when structural biological features of this protein are limiting.

Our data suggest that inhibition of ANO1-dependent chloride conductance is not sufficient to promote degradation of ANO1. T16A_inh_-A01 and digallic acid had no effect on the proliferation of ANO1-dependent cells and did not reduce protein levels of ANO1, although both compounds inhibited ANO1-dependent chloride conductance with a similar potency as CaCC_inh_-A01 (17, 18, 30). T16A_inh_-A01 is a reported inhibitor of ANO1-dependent cell proliferation (15, 42). However, these experiments were performed at high concentrations of compound and resulted in weak effects on proliferation. These data suggest that inhibition of ANO1 channel activity alone is not sufficient to inhibit cell proliferation and that the reduction of ANO1 protein levels is requisite. ANO1 has been shown to interact with cytoskeletal proteins and to promote epidermal growth factor receptor/calcium/calmodulin-dependent protein kinase signaling in cancer cells (6, 15, 16), yet a detailed understanding of the involvement of ANO1 in these pathways remains unclear. A recent study in dorsal root ganglia found ANO1 to be in a complex with the inositol 1,4,5-trisphosphate receptor 1, bradykinin receptor 2, the protease-activated receptor 2, and caveolin and to be tethered to juxtamembrane regions of the ER and suggests that a similar complex may exist in cancer cells (43). Future studies may show whether the compounds described herein may interfere with the complex, resulting in the destabilization and degradation of ANO1.

Using our combined experimental and computational techniques, we have made progress toward identification of potent and selective ANO1 inhibitors and uncovering the mechanism of how these inhibitors may work in cells. Our results provide impetus for gaining a deeper understanding of ANO1 modulation in cells, which could impact the treatment of ANO1-amplified cancers.

## Footnotes

3 The abbreviations used are:

HNSCC: head and neck squamous carcinoma
ESCC: esophageal squamous carcinoma
CaCC: calcium-activated chloride channel
ER: endoplasmic reticulum
PGG: pentagalloyl glucose
BFA: brefeldin A.

## References

[1] Jonkers J. Berns A. (2004) Oncogene addiction: sometimes a temporary slavery. Cancer Cell 6, 535–8 15607957 10.1016/j.ccr.2004.12.002

[2] West R. B. Corless C. L. Chen X. Rubin B. P. Subramanian S. Montgomery K. Zhu S. Ball C. A. Nielsen T. O. Patel R. Goldblum J. R. Brown P. O. Heinrich M. C. van de Rijn M. (2004) The novel marker, DOG1, is expressed ubiquitously in gastrointestinal stromal tumors irrespective of KIT or PDGFRA mutation status. Am. J. Pathol. 165, 107–113 15215166 10.1016/S0002-9440(10)63279-8 PMC1618538

[3] Kashyap M. K. Marimuthu A. Kishore C. J. Peri S. Keerthikumar S. Prasad T. S. Mahmood R. Rao S. Ranganathan P. Sanjeeviah R. C. Vijayakumar M. Kumar K. V. Montgomery E. A. Kumar R. V. Pandey A. (2009) Genomewide mRNA profiling of esophageal squamous cell carcinoma for identification of cancer biomarkers. Cancer Biol. Ther. 8, 36–46 18981721 10.4161/cbt.8.1.7090

[4] Carles A. Millon R. Cromer A. Ganguli G. Lemaire F. Young J. Wasylyk C. Muller D. Schultz I. Rabouel Y. Dembélé D. Zhao C. Marchal P. Ducray C. Bracco L. Abecassis J. Poch O. Wasylyk B. (2006) Head and neck squamous cell carcinoma transcriptome analysis by comprehensive validated differential display. Oncogene 25, 1821–1831 16261155 10.1038/sj.onc.1209203

[5] Huang X. Gollin S. M. Raja S. Godfrey T. E. (2002) High-resolution mapping of the 11q13 amplicon and identification of a gene, *TAOS1*, that is amplified and overexpressed in oral cancer cells. Proc. Natl. Acad. Sci. U.S.A. 99, 11369–11374 12172009 10.1073/pnas.172285799 PMC123263

[6] Britschgi A. Bill A. Brinkhaus H. Rothwell C. Clay I. Duss S. Rebhan M. Raman P. Guy C. T. Wetzel K. George E. Popa M. O. Lilley S. Choudhury H. Gosling M. Wang L. Fitzgerald S. Borawski J. Baffoe J. Labow M. Gaither L. A. Bentires-Alj M. (2013) Calcium-activated chloride channel ANO1 promotes breast cancer progression by activating EGFR and CAMK signaling. Proc. Natl. Acad. Sci. U.S.A. 110, E1026–E1034 23431153 10.1073/pnas.1217072110 PMC3600458

[7] Shi Z. Z. Shang L. Jiang Y. Y. Hao J. J. Zhang Y. Zhang T. T. Lin D. C. Liu S. G. Wang B. S. Gong T. Zhan Q. M. Wang M. R. (2013) Consistent and differential genetic aberrations between esophageal dysplasia and squamous cell carcinoma detected by array comparative genomic hybridization. Clin. Cancer Res. 19, 5867–5878 24009147 10.1158/1078-0432.CCR-12-3753

[8] Yang Y. D. Cho H. Koo J. Y. Tak M. H. Cho Y. Shim W. S. Park S. P. Lee J. Lee B. Kim B. M. Raouf R. Shin Y. K. Oh U. (2008) TMEM16A confers receptor-activated calcium-dependent chloride conductance. Nature 455, 1210–1215 18724360 10.1038/nature07313

[9] Schroeder B. C. Cheng T. Jan Y. N. Jan L. Y. (2008) Expression cloning of TMEM16A as a calcium-activated chloride channel subunit. Cell 134, 1019–1029 18805094 10.1016/j.cell.2008.09.003 PMC2651354

[10] Caputo A. Caci E. Ferrera L. Pedemonte N. Barsanti C. Sondo E. Pfeffer U. Ravazzolo R. Zegarra-Moran O. Galietta L. J. (2008) TMEM16A, a membrane protein associated with calcium-dependent chloride channel activity. Science 322, 590–594 18772398 10.1126/science.1163518

[11] Huang F. Wong X. Jan L. Y. (2012) International Union of Basic and Clinical Pharmacology. LXXXV: calcium-activated chloride channels. Pharmacol. Rev. 64, 1–15 22090471 10.1124/pr.111.005009 PMC3250081

[12] Cho H. Yang Y. D. Lee J. Lee B. Kim T. Jang Y. Back S. K. Na H. S. Harfe B. D. Wang F. Raouf R. Wood J. N. Oh U. (2012) The calcium-activated chloride channel anoctamin 1 acts as a heat sensor in nociceptive neurons. Nat. Neurosci. 15, 1015–1021 22634729 10.1038/nn.3111

[13] Zhang C. H. Li Y. Zhao W. Lifshitz L. M. Li H. Harfe B. D. Zhu M. S. ZhuGe R. (2013) The TMEM16A Ca^2+^-activated Cl^−^ channel in airway smooth muscle contributes to airway hyperresponsiveness. Am. J. Respir. Crit. Care Med. 187, 374–381 23239156 10.1164/rccm.201207-1303OC PMC3603598

[14] Rock J. R. Futtner C. R. Harfe B. D. (2008) The transmembrane protein TMEM16A is required for normal development of the murine trachea. Dev. Biol. 321, 141–149 18585372 10.1016/j.ydbio.2008.06.009

[15] Duvvuri U. Shiwarski D. J. Xiao D. Bertrand C. Huang X. Edinger R. S. Rock J. R. Harfe B. D. Henson B. J. Kunzelmann K. Schreiber R. Seethala R. S. Egloff A. M. Chen X. Lui V. W. Grandis J. R. Gollin S. M. (2012) TMEM16A induces MAPK and contributes directly to tumorigenesis and cancer progression. Cancer Res. 72, 3270–3281 22564524 10.1158/0008-5472.CAN-12-0475-T PMC3694774

[16] Perez-Cornejo P. Gokhale A. Duran C. Cui Y. Xiao Q. Hartzell H. C. Faundez V. (2012) Anoctamin 1 (Tmem16A) Ca^2+^-activated chloride channel stoichiometrically interacts with an ezrin-radixin-moesin network. Proc. Natl. Acad. Sci. U.S.A. 109, 10376–10381 22685202 10.1073/pnas.1200174109 PMC3387097

[17] De La Fuente R. Namkung W. Mills A. Verkman A. S. (2008) Small-molecule screen identifies inhibitors of a human intestinal calcium-activated chloride channel. Mol. Pharmacol. 73, 758–68 18083779 10.1124/mol.107.043208

[18] Namkung W. Phuan P. W. Verkman A. S. (2011) TMEM16A inhibitors reveal TMEM16A as a minor component of calcium-activated chloride channel conductance in airway and intestinal epithelial cells. J. Biol. Chem. 286, 2365–2374 21084298 10.1074/jbc.M110.175109 PMC3023530

[19] Kumar S. Namkung W. Verkman A. S. Sharma P. K. (2012) Novel 5-substituted benzyloxy-2-arylbenzofuran-3-carboxylic acids as calcium activated chloride channel inhibitors. Bioorg. Med. Chem. 20, 4237–4244 22739085 10.1016/j.bmc.2012.05.074 PMC3643516

[20] Huang F. Zhang H. Wu M. Yang H. Kudo M. Peters C. J. Woodruff P. G. Solberg O. D. Donne M. L. Huang X. Sheppard D. Fahy J. V. Wolters P. J. Hogan B. L. Finkbeiner W. E. Li M. Jan Y. N. Jan L. Y. Rock J. R. (2012) Calcium-activated chloride channel TMEM16A modulates mucin secretion and airway smooth muscle contraction. Proc. Natl. Acad. Sci. U.S.A. 109, 16354–16359 22988107 10.1073/pnas.1214596109 PMC3479591

[21] Cheeseright T. Mackey M. Rose S. Vinter A. (2006) Molecular field extrema as descriptors of biological activity: definition and validation. J. Chem. Inf. Model. 46, 665–676 16562997 10.1021/ci050357s

[22] Cheeseright T. J. Mackey M. D. Melville J. L. Vinter J. G. (2008) FieldScreen: virtual screening using molecular fields. Application to the DUD data set. J. Chem. Inf. Model. 48, 2108–17 18991371 10.1021/ci800110p

[23] Duan J. Dixon S. L. Lowrie J. F. Sherman W. (2010) Analysis and comparison of 2D fingerprints: insights into database screening performance using eight fingerprint methods. J. Mol. Graph. Model. 29, 157–170 20579912 10.1016/j.jmgm.2010.05.008

[24] Sastry M. Lowrie J. F. Dixon S. L. Sherman W. (2010) Large-scale systematic analysis of 2D fingerprint methods and parameters to improve virtual screening enrichments. J. Chem. Inf. Model. 50, 771–784 20450209 10.1021/ci100062n

[25] Fallah G. Romer T. Detro-Dassen S. Braam U. Markwardt F. Schmalzing G. (2011) TMEM16A(a)/anoctamin-1 shares a homodimeric architecture with CLC chloride channels. Mol. Cell Proteomics 10, M110.004697 20974900 10.1074/mcp.M110.004697 PMC3033684

[26] Sheridan J. T. Worthington E. N. Yu K. Gabriel S. E. Hartzell H. C. Tarran R. (2011) Characterization of the oligomeric structure of the Ca^2+^-activated Cl^−^ channel Ano1/TMEM16A. J. Biol. Chem. 286, 1381–1388 21056985 10.1074/jbc.M110.174847 PMC3020746

[27] Bill A. Schmitz A. Albertoni B. Song J. N. Heukamp L. C. Walrafen D. Thorwirth F. Verveer P. J. Zimmer S. Meffert L. Schreiber A. Chatterjee S. Thomas R. K. Ullrich R. T. Lang T. Famulok M. (2010) Cytohesins are cytoplasmic ErbB receptor activators. Cell 143, 201–211 20946980 10.1016/j.cell.2010.09.011

[28] Mathes C Friis S Finley M Liu Y. (2009) QPatch: the missing link between HTS and ion channel drug discovery. Comb. Chem. High Throughput Screen. 12, 78–95 19149494 10.2174/138620709787047948

[29] Tian Y. Schreiber R. Kunzelmann K. (2012) Anoctamins are a family of Ca^2+^ activated Cl^−^ channels. J. Cell Sci. 125, 4991–4998 22946059 10.1242/jcs.109553

[30] Namkung W. Thiagarajah J. R. Phuan P. W. Verkman A. S. (2010) Inhibition of Ca^2+^-activated Cl^−^ channels by gallotannins as a possible molecular basis for health benefits of red wine and green tea. FASEB J. 24, 4178–4186 20581223 10.1096/fj.10-160648 PMC2974422

[31] Zhang J. Li L. Kim S. H. Hagerman A. E. Lü J. (2009) Anti-cancer, anti-diabetic and other pharmacologic and biological activities of penta-galloyl-glucose. Pharm. Res. 26, 2066–2080 19575286 10.1007/s11095-009-9932-0 PMC2822717

[32] Tien J. Lee H. Y. Minor D. L. Jr. Jan Y. N. Jan L. Y. (2013) Identification of a dimerization domain in the TMEM16A calcium-activated chloride channel (CaCC). Proc. Natl. Acad. Sci. U.S.A. 110, 6352–6357 23576756 10.1073/pnas.1303672110 PMC3631655

[33] Misumi Y. Misumi Y. Miki K. Takatsuki A. Tamura G. Ikehara Y. (1986) Novel blockade by brefeldin A of intracellular transport of secretory proteins in cultured rat hepatocytes. J. Biol. Chem. 261, 11398–11403 2426273

[34] Lippincott-Schwartz J. Donaldson J. G. Schweizer A. Berger E. G. Hauri H. P. Yuan L. C. Klausner R. D. (1990) Microtubule-dependent retrograde transport of proteins into the ER in the presence of brefeldin A suggests an ER recycling pathway. Cell 60, 821–836 2178778 10.1016/0092-8674(90)90096-w

[35] Abriel H. Staub O. (2005) Ubiquitylation of ion channels. Physiology 20, 398–407 16287989 10.1152/physiol.00033.2005

[36] Claessen J. H. Kundrat L. Ploegh H. L. (2012) Protein quality control in the ER: balancing the ubiquitin checkbook. Trends Cell Biol. 22, 22–32 22055166 10.1016/j.tcb.2011.09.010 PMC3564647

[37] Dalemans W. Barbry P. Champigny G. Jallat S. Dott K. Dreyer D. Crystal R. G. Pavirani A. Lecocq J. P. Lazdunski M. (1991) Altered chloride ion channel kinetics associated with the Δ F508 cystic fibrosis mutation. Nature 354, 526–528 1722027 10.1038/354526a0

[38] Ward C. L. Omura S. Kopito R. R. (1995) Degradation of CFTR by the ubiquitin-proteasome pathway. Cell 83, 121–127 7553863 10.1016/0092-8674(95)90240-6

[39] Tian Y. Schreiber R. Wanitchakool P. Kongsuphol P. Sousa M. Uliyakina I. Palma M. Faria D. Traynor-Kaplan A. E. Fragata J. I. Amaral M. D. Kunzelmann K. (2013) Control of TMEM16A by INO-4995 and other inositolphosphates. Br. J. Pharmacol. 168, 253–265 22946960 10.1111/j.1476-5381.2012.02193.x PMC3570019

[40] Lins L. Brasseur R. (1995) The hydrophobic effect in protein folding. FASEB J. 9, 535–540 7737462 10.1096/fasebj.9.7.7737462

[41] Neklesa T. K. Tae H. S. Schneekloth A. R. Stulberg M. J. Corson T. W. Sundberg T. B. Raina K. Holley S. A. Crews C. M. (2011) Small-molecule hydrophobic tagging-induced degradation of HaloTag fusion proteins. Nat. Chem. Biol. 7, 538–543 21725302 10.1038/nchembio.597 PMC3139752

[42] Mazzone A. Eisenman S. T. Strege P. R. Yao Z. Ordog T. Gibbons S. J. Farrugia G. (2012) Inhibition of cell proliferation by a selective inhibitor of the Ca^2+^-activated Cl^−^ channel, Ano1. Biochem. Biophys. Res. Commun. 427, 248–253 22995309 10.1016/j.bbrc.2012.09.022 PMC3479349

[43] Jin X. Shah S. Liu Y. Zhang H. Lees M. Fu Z. Lippiat J. D. Beech D. J. Sivaprasadarao A. Baldwin S. A. Zhang H. Gamper N. (2013) Activation of the Cl^−^ channel ANO1 by localized calcium signals in nociceptive sensory neurons requires coupling with the IP_3_ receptor. Sci. Signal. 6, ra73 23982204 10.1126/scisignal.2004184 PMC4135425

